# Neuron type-specific miRNA represses two broadly expressed genes to modulate an avoidance behavior in *C. elegans*

**DOI:** 10.1101/gad.287904.116

**Published:** 2016-09-15

**Authors:** Tanja Drexel, Katharina Mahofsky, Richard Latham, Manuel Zimmer, Luisa Cochella

**Affiliations:** Research Institute of Molecular Pathology (IMP), Vienna Biocenter (VBC), 1030 Vienna, Austria

**Keywords:** miRNAs, cell identity, *C. elegans*, carbon dioxide

## Abstract

Two broad gene classes are distinguished within multicellular organisms: cell type-specific genes, which confer particular cellular properties, and ubiquitous genes that support general cellular functions. However, certain so-called ubiquitous genes show functionally relevant cell type-specific repression. How such repression is achieved is poorly understood. MicroRNAs (miRNAs) are repressors, many of which are expressed with high cell type specificity. Here we show that *mir-791*, expressed exclusively in the CO_2_-sensing neurons in *Caenorhabditis elegans*, represses two otherwise broadly expressed genes. This repression is necessary for normal neuronal function and behavior of the animals toward CO_2_. miRNA-mediated repression of broadly transcribed genes is a previously unappreciated strategy for cellular specialization.

## Abstract (precis)

In this study, Drexel et al. research miRNA-mediated repression of broadly transcribed genes as a strategy for cellular specialization. They show that mir-791, expressed exclusively in the CO_2_-sensing neurons in *C. elegans*, represses two otherwise broadly expressed genes, which are needed for normal neuronal function and behavior of the animals toward CO_2_.

The ability to regulate different sets of genes to generate and maintain distinct cell types with diverse morphologies and functions is the basis for multicellularity. Evolutionary studies suggest that this ability derived from an ancestral multifunctional eukaryotic cell expressing a relatively high number of genes. Evolution to multicellularity relied on the segregation of functions from that primordial cell into an increasingly diversified number of descendants (Arendt 2008). Functional segregation as well as acquisition of new cell-specific functions resulted in genes expressed in specific cell types, while others remained broadly or even ubiquitously expressed. The addition of repressors of gene expression must have been key during this process, restricting the execution of genetic programs to specific cell types.

It has long been recognized that even so-called ubiquitous genes are regulated in specific cells or conditions. For example, the housekeeping gene encoding the lactate/pyruvate transporter MCT1 is specifically repressed in pancreatic β cells to achieve a correct cellular and organismal response to changes in glucose concentration (Ishihara et al. 1999). However, mechanisms for cell-specific repression of broadly expressed genes remain relatively unexplored.

MicroRNAs (miRNAs) are a broad class of post-transcriptional repressors that expanded with the onset of multicellularity (Berezikov 2011). Since miRNA evolution is more dynamic than that of protein-coding genes and since many miRNAs are expressed with high spatiotemporal specificity (e.g., see Aboobaker et al. 2005; Wienholds et al. 2005), they are proposed to contribute to the gene expression profiles of specific cell types. While several examples illustrate the roles of miRNAs in refining expression patterns of other spatiotemporally restricted genes, broadly expressed housekeeping genes tend to avoid miRNA-binding sites in their 3′ untranslated regions (UTRs) (Stark et al. 2005). However, it is conceivable that miRNAs present exclusively in a given cell type can repress broadly transcribed genes in those cells. Specific repression could provide unique features to specialized cells while preserving the function of such broadly transcribed targets in the rest of the organism. Moreover, post-transcriptional regulation provides an appealing solution to regulate housekeeping genes, whose transcription is constrained by the compact nature of their promoters (Zeitlinger and Stark 2010; Zabidi et al. 2015).

Supporting this hypothesis, we show that *mir-791* is expressed exclusively in three pairs of sensory neurons in the nematode *Caenorhabditis elegans* that are essential for the avoidance response to high CO_2_ (Bretscher et al. 2011). We found that *mir-791* represses two target genes that are transcribed in all types of somatic cells, consistent with their proposed functions in general cell biology: a PKA anchor protein (*akap-1* [*A kinase anchor protein 1*]) and a carbonic anhydrase (*cah-3*). miRNA-mediated repression of these targets in the CO_2_-sensing neurons is necessary for normal neuronal physiology and, ultimately, the animal's response to this critical environmental cue.

## Results and Discussion

### *mir-791* is necessary for a normal response of *C. elegans* to CO_2_

**Figure 1.**
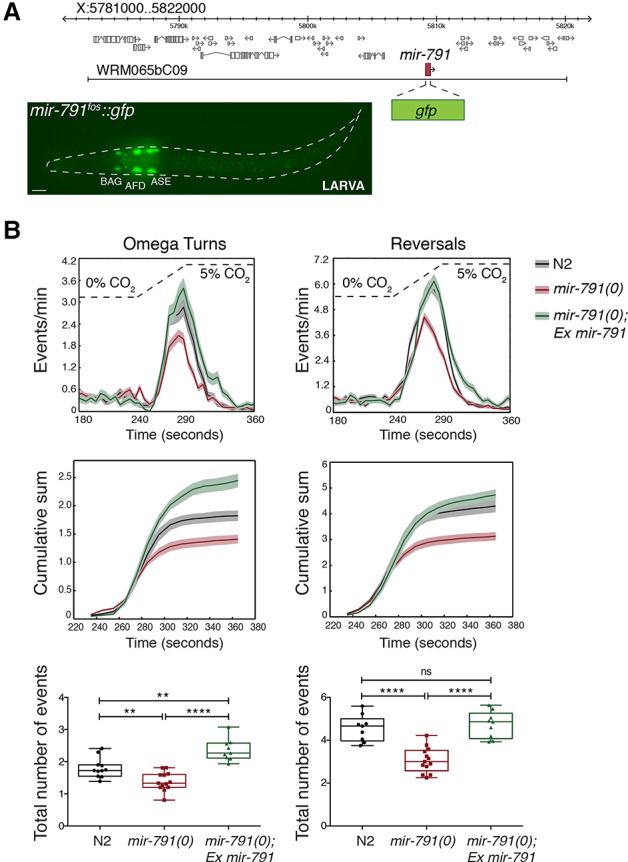
*mir-791* is expressed in the CO_2_-sensing neurons of *C. elegans* and is required for the response to CO_2_. (*A*) Schematic of the fosmid-based reporter used to monitor *mir-791* expression and a representative image of a transgenic animal; the three pairs of neurons are labeled. Bar, 10 μm. (*B*, *top*) Population means (lines) and standard errors of the means (shading) of turning and reversal rates for wild type (N2), *mir-791(0)*, and *mir-791(0)* with extrachromosomal copies of *mir-791* during a gradual increase of CO_2_ from 0% to 5%. (*Middle*) Cumulative sum of all of the turning or reversal events during the stimulation phase (240–360 sec). (*Bottom*) Box and whiskers representation of the total number of turns and reversals during the stimulation phase (each data point is an endpoint of the individual replicate used to calculate the averages shown *above*). *n* = 9 N2; *n* = 13 *mir-791(0)*; *n* = 9 *mir-791(0), Ex mir-791*. Each replicate is an experiment with 50–100 animals. Boxes show interquartile range, and whiskers indicate full range. (****) *P* < 0.0001; (***) *P* < 0.001; (**) *P* < 0.01; (*) *P* < 0.05; (ns) not significant, Mann-Whitney test.

We conducted a screen for miRNAs present in specific *C. elegans* neurons using reporters with ∼40 kb of genomic sequence in fosmid vectors, which accurately recapitulate endogenous expression patterns (Tursun et al. 2009). We found *mir-791* exclusively in three pairs of sensory neurons, which, based on position, morphology, and expression of known markers, were identified as the BAG, AFD, and ASE pairs of neurons (Fig. 1A; Supplemental Fig. S1A). These neurons are the main cells responsible for sensing elevated CO_2_ and triggering the escape response in *C. elegans* (Bretscher et al. 2011), as high CO_2_ is toxic and is proposed to signal the presence of predators (Bretscher et al. 2008; Hallem and Sternberg 2008; Sharabi et al. 2009). Expression of *mir-791* begins in embryos shortly after the neurons’ birth and continues throughout adulthood (Supplemental Fig. S1A; our small RNA sequencing data not shown). Given the specificity of *mir-791* expression, we hypothesized that it may be involved in CO_2_ sensing in *C. elegans*.

We used the escape response of *C. elegans* from high CO_2_ as a proxy for the ability of the worms to sense changes in CO_2_ levels (Bretscher et al. 2008; Hallem and Sternberg 2008). *C. elegans* responds to diverse stimuli by modulating the frequency of reorientation maneuvers (Pierce-Shimomura et al. 1999), such as switching from forward to backward-directed crawling (reversal) and performing sharp-angle turns (Ω turn). Worms use a single reversal–turning sequence to acutely escape aversive stimuli (Chalfie et al. 1985). To test the role of *mir-791* in the CO_2_-evoked escape behavior, we deleted the miRNA locus using CRISPR/Cas9 and compared these animals with the wild type in their response to a gradual increase of CO_2_ from 0% to 5%. Upon this stimulus, wild-type animals robustly increase their frequency of reversals and turns. Animals lacking *mir-791* display a significant reduction in these maneuvers in response to the same stimulus (Fig. 1B), while their average speed modulation is the same as wild type (Supplemental Fig. S2). *mir-791-*deficient animals have a similar onset of response to CO_2_ but stop responding to the stimulus before their wild-type counterparts, an effect best visualized by displaying the cumulative sum of turns or reversals over time (Fig. 1B, middle). To confirm that this effect is due to loss of *mir-791*, we reintroduced *mir-791* under its endogenous promoter as an extrachromosomal multicopy transgene and found that this rescued the worm's response to CO_2_ (Fig. 1B). In fact, it is likely that overexpression of *mir-791* from this transgene increased the locomotor response of these animals to levels higher than wild type.

*mir-791* has been linked in a family with *mir-790* due to their identical seed sequences (Supplemental Fig. S1B). In addition, *mir-790* is also expressed in the CO_2_-sensing neurons (Supplemental Fig. S1C), suggesting that they could act redundantly. However, using a deletion allele that we generated for *mir-790* by itself or in a double-mutant combination with *mir-791(0)*, we showed that this is not the case (Supplemental Fig. S1D–F). Overall, we found that a single miRNA, *mir-791*, which is expressed exclusively in the CO_2_-sensing neurons of *C. elegans*, is required for the normal behavioral response of the animals to CO_2_.

### *mir-791* is required mainly in the BAGs and functions specifically in CO_2_ sensing

While *mir-791* is transcribed in three pairs of CO_2_-sensing neurons, the BAGs have the highest contribution to the avoidance behavior (Bretscher et al. 2011). To test whether the role of *mir-791* was more significant in BAG than in AFD or ASE, we expressed *mir-791* under promoters specific for each of these neurons in *mir-791(0)* animals. Reintroduction of *mir-791* in BAG alone was sufficient to recover the response to elevated CO_2_ to a large extent, while exclusive expression in AFD or ASE alone did not rescue the *mir-791(0)* defect (Supplemental Fig. S3). *mir-791* likely contributes to all three neuron pairs, but its role in the BAG neurons accounts for the largest fraction of the measurable phenotype. Hence, we focused on these neurons for further characterization.

To test whether *mir-791* acts specifically in CO_2_ sensing or more generally affects BAG function, we took advantage of the fact that these neurons also sense decreases in O_2_ concentration (Zimmer et al. 2009). Using the same behavioral setup, we asked whether *mir-791(0)* animals have a defect in their response to changes in O_2_ while keeping CO_2_ at 0%. In this assay, animals lacking *mir-791* respond to O_2_ changes indistinguishably from wild type (Supplemental Fig. S4). We conclude that *mir-791* plays a specific role in CO_2_ sensing and is not generally required for other sensory functions of the BAGs. Also, as *mir-791(0)* animals show normal basal speed in the absence of stimuli and since normal frequency of turns and reversals upon O_2_ decreases, we ruled out a general locomotion defect in these animals.

### *mir-791* is not required for the basic cellular identity of the BAGs but is required for neuronal physiology

**Figure 2.**
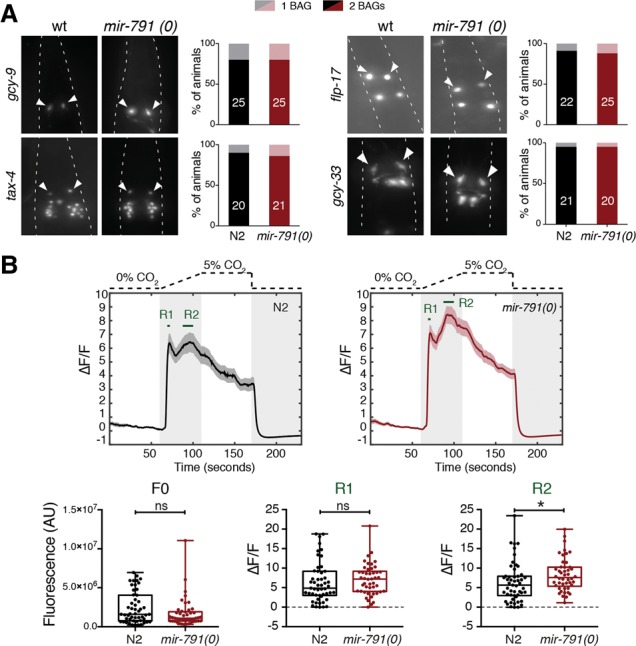
*mir-791* is not required for expression of core BAG genes but is necessary for a wild-type response to CO_2_. (*A*) Representative fluorescence images of animals expressing reporters for functionally important genes in the BAG neurons in wild type (wt; N2) and *mir-791(0)*. For the transgenes used, see Supplemental Table S2. Twenty or more animals of each genotype were scored for the presence of each reporter (shown in the bar graphs). (*B*) Mean and SEM of changes in fluorescence of GCaMP6f in the BAG neurons in response to changes in CO_2_. *n* = 51 N2; *n* = 46 *mir-791(0)*. Statistics (Mann-Whitney test) on the baseline (F0), initial (R1), and sustained (R2) responses are shown.

As *mir-791* is expressed from the time the CO_2_ sensory neurons are born, we asked whether it plays a role in specifying the identity of these cells. To do so, we scored the expression of functionally relevant terminal markers of these neurons in animals with or without *mir-791*. The candidate CO_2_ sensor in the BAGs is a receptor guanylate cyclase encoded by *gcy-9* (Hallem et al. 2011; Smith et al. 2013). Upon CO_2_ increase, GCY-9 is thought to generate cGMP, opening a cGMP-gated channel formed by the products of *tax-2* and *tax-4.* We generated a fosmid-based reporter for *gcy-9* and used available reporters for *tax-4* and also *gcy-33*, a soluble guanylate cyclase involved in O_2_ sensing (Zimmer et al. 2009), and *flp-17*, a neuropeptide secreted by the BAGs (Ringstad and Horvitz 2008). All of these reporters were expressed indistinguishably in the BAG neurons in the presence or absence of *mir-791* (Fig. 2A). Also, the morphology and position of the neurons were unaffected. This is in line with the partial decrease in the behavioral response to CO_2_ in *mir-791(0)* animals as compared with animals in which the BAGs are ablated (Bretscher et al. 2011). Together, this supports that *mir-791* is not required for specifying the basic identity of the BAGs and implies that targets of *mir-791* might impact the physiology of these cells.

To monitor the physiological response of the BAGs, we measured their CO_2_-evoked activity using the genetically encoded fluorescent calcium indicator GCaMP6f (Chen et al. 2013). Upon the same CO_2_ increase as above, the BAG neurons responded reliably in wild-type animals with a biphasic profile of activity followed by a gradual decline even though CO_2_ was still high, suggesting that the neurons become desensitized to the stimulus (Fig. 2B). Animals lacking *mir-791* had a similar initial response but a slightly higher and more sustained second phase of activity (Fig. 2B; Supplemental Fig. S5). The biphasic increase in fluorescence reflects the BAG response to CO_2_, as both phases are abolished in animals lacking the CO_2_ sensor GCY-9 (Supplemental Fig. S5C). While we cannot rule out additional effects downstream from Ca^2+^ influx, this suggests that *mir-791* has an effect on the Ca^2+^ response of the BAGs to CO_2_. Whether this differential response explains the behavioral defect remains to be tested, but it suggests the possibility that sustained BAG activity may somehow limit the measured behavioral response.

### *akap-1* and *cah-3* are broadly expressed but are specifically repressed by *mir-791* in the BAGs

TargetScanWorm 6.2 (Jan et al. 2011) provided us with a short list of likely *mir-791* targets. Among the top 10 predicted targets, there were five genes with multiple *mir-791-*binding sites whose protein products suggested possible links to CO_2_ sensing or neuronal signaling (Supplemental Table S1).

The top two predicted targets were particularly interesting. AKAP-1 is a member of a protein family initially found to tether the cAMP-dependent kinase PKA to distinct cellular compartments, generating subcellular signaling domains. However, AKAPs also bind phosphatases, phosphodiesterases, and other regulators of cyclic nucleotide signaling (Langeberg and Scott 2015). We hypothesized that AKAP-1 could affect cyclic nucleotide signaling downstream from GCY-9. The second predicted target, *cah-3*, encodes a carbonic anhydrase (CA). These enzymes catalyze the reversible hydration of CO_2_ to produce HCO_3_^−^ and H^+^ and are essential to maintain CO_2_, electrolyte, and pH homeostasis (Supuran 2008). In addition, CAs have been implicated in CO_2_ sensing in multiple systems (Luo et al. 2009; Cummins et al. 2013).

**Figure 3.**
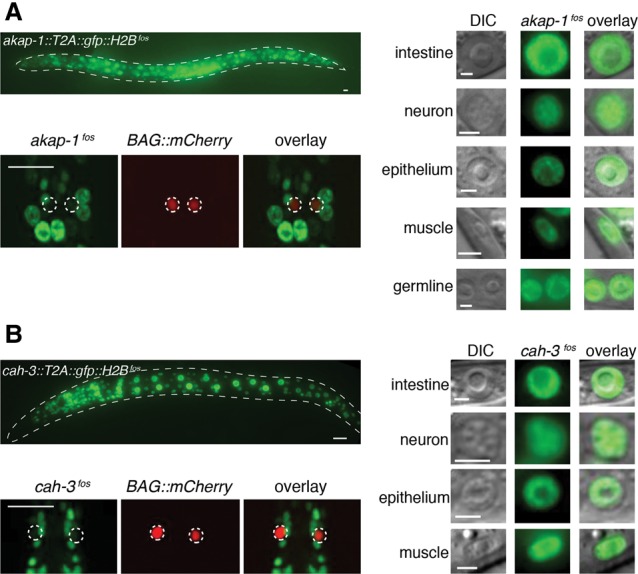
AKAP-1 and CAH-3 are expressed in most cell types of the animal but are low or absent in the BAG neurons. Representative fluorescence images of animals carrying *akap-1* (*A*) and *cah-3* (*B*) nuclear-localized fosmid-based reporters (Supplemental Fig. S6). (*Right*) Zoom in of the different nuclear types (distinguished by size and morphology) expressing both reporters. Bars, 2 μm. (*Bottom left*) Focus on the BAG nuclei localized with *flp-17^prom^:NLS:mCherry*. Both reporters are very low or absent in these cells. Bars, 10 μm.

To investigate the expression of these genes, we generated fosmid-based fluorescent reporters (Supplemental Fig. S6; Tursun et al. 2009). To facilitate visualization and quantification, we inserted a T2A peptide followed by GFP:H2B at the C terminus of each of the genes such that two independent polypeptides were made: untagged AKAP-1 or CAH-3 and nuclear GFP:H2B. Production of both proteins is under regulation by the wild-type 3′ UTR. Both reporters are broadly expressed in most tissues of worms. *akap-1* is expressed from early embryos until adulthood and in most cells of the animal, both soma and germline (Fig. 3A; Supplemental Fig. S6A). Its homolog in *Drosophila*, *spoonbill*, is also ubiquitously expressed in flies of all stages (http://www.flybase.org; http://www.fruitfly.org). The *cah-3* reporter is also broadly expressed in all major somatic cell types (Fig. 3B; Supplemental Fig. S6B). The broad expression of these two genes is in line with their proposed general cellular functions.

**Figure 4.**
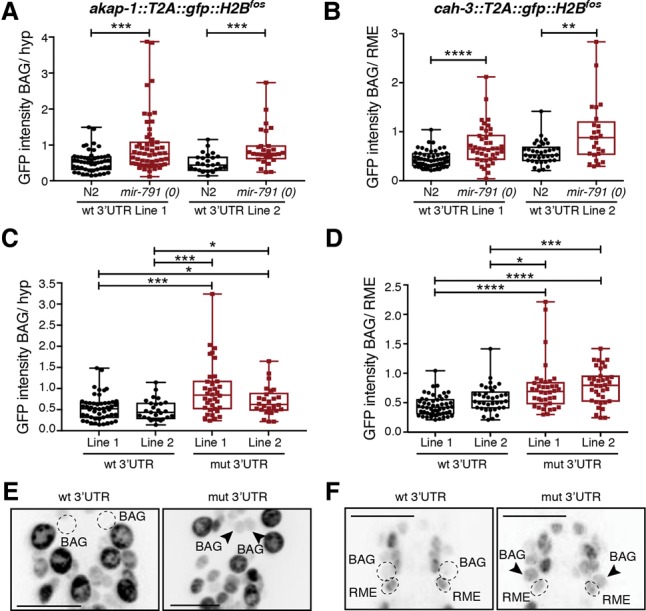
*mir-791* represses *akap-1* and *cah-3* specifically in the BAG neurons. (*A*,*B*) Normalized GFP intensity in the BAG neurons of animals carrying *akap-1* or *cah-3* fosmid reporters with their respective wild-type 3′ UTRs in either wild-type (N2) or *mir-791(0)* animals. Two independent lines were scored per reporter. (*C*,*D*) Normalized GFP intensity in the BAG neurons of animals carrying *akap-1* or *cah-3* fosmid reporters with either wild-type or mutant 3′ UTRs (without *mir-791-*binding sites) (Supplemental Fig. S6C). Asterisks show *P*-values (Mann-Whitney test) as in Figure 1. (*E*,*F*) Representative images of the GFP expression of the reporters quantified in *C* and *D*. Inverted images are shown. BAG nuclei were localized with *flp-17^prom^:NLS:mCherry* (not shown); their position is marked by a circle when GFP expression is not visible or arrowheads otherwise. For details on quantification, see the Supplemental Material. Bars, 10 μm.

In the BAG neurons, however, GFP fluorescence from both reporters was barely detectable (Fig. 3). To test whether the low-level expression of *cah-3* and *akap-1* in the BAGs is due to *mir-791*, we measured GFP intensity in these cells in wild-type or *mir-791(0)* backgrounds. The absence of *mir-791* resulted in the derepression of both *akap-1* and *cah-3* reporters in the BAGs to levels similar to neighboring reference cells that do not express *mir-791* (Fig. 4A,B). In addition, removing all seed-matching sequences in the 3′ UTRs of both reporters (Supplemental Fig. S6C) also caused derepression of the *akap-1* and *cah-3* reporters to a similar extent (Fig. 4C–F). These experiments show that while *akap-1* and *cah-3* are broadly transcribed, *mir-791* specifically represses these genes in the BAG neurons.

### *akap-1* and *cah-3* are functional targets of *mir-791*

To test whether these genes are true functional targets of *mir-791*, we introduced 3′ UTR mutations to disrupt all predicted *mir-791-*binding sites in the endogenous *akap-1* or *cah-3* loci (as in Supplemental Fig. S6C) and also in the other three top predicted targets. We did this by CRISPR/Cas9-induced homology-directed repair. If any of these genes is a relevant target of *mir-791*, removal of the binding sites should recapitulate, at least in part, the defect caused by removal of *mir-791* itself.

**Figure 5.**
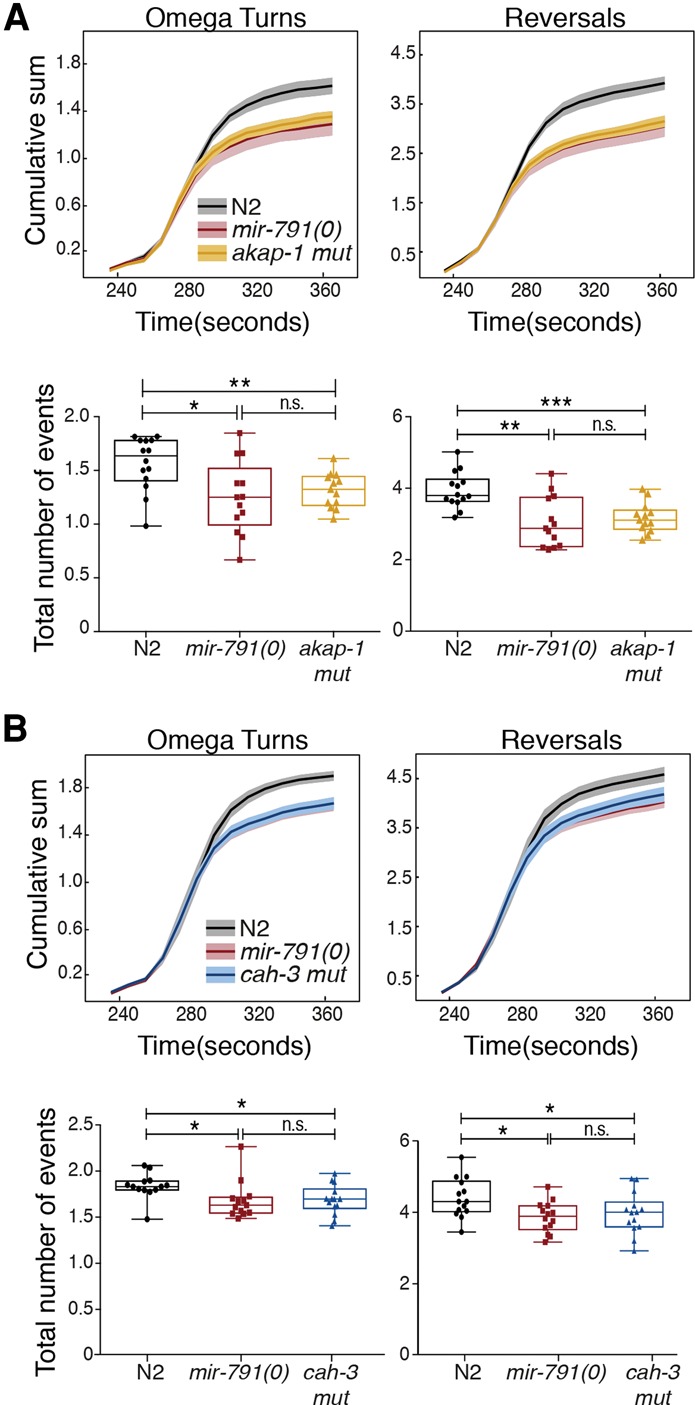
*akap-1* and *cah-3* regulation accounts for the observed *mir-791(0)* phenotype. (*A*) Cumulative sum plots and statistical analysis of turning and reversal events (as in Fig. 1) of wild-type (N2) (*n* = 14), *mir-791(0)* (*n* = 13), and *akap-1* 3′ UTR mutant (*n* = 13) animals. (*B*) Same as in *A* but showing comparison with *cah-3* 3′ UTR mutant animals. *n* = 14 wild-type (N2); *n* = 14 *mir-791(0)*; *n* = 15 *cah-3* 3′ UTR mutant.

Disrupting the *mir-791-*binding sites in *akap-1* or *cah-3* phenocopied the loss of *mir-791* in the CO_2_ response assay (Fig. 5). However, mutations in the 3′ UTRs of *hbl-1*, *unc-9*, and *unc-2* did not affect this behavioral response (Supplemental Fig. S7A–C). We also generated a strain with all five 3′ UTR mutations. These animals display a behavioral defect similar to *mir-791(0)* (Supplemental Fig. S7D). These data strongly support *akap-1* and *cah-3* as the two critical targets of *mir-791* in the CO_2_-sensing neurons. In addition, the fact that each target seems to fully account for the effect of *mir-791* suggests that they act in the same genetic pathway.

At the molecular level, a higher dose of CAH-3 will affect the kinetics of CO_2_ and HCO_3_^−^ interconversion and also likely the local intracellular pH. This might have an impact on signaling through GCY-9 or downstream events. AKAP1 has been shown to generate signaling hubs on the mitochondrial surface (Merrill and Strack 2014). We thus speculate that high AKAP-1 levels in the BAGs might ectopically tether required signaling components away from their site of action.

CAs are expressed in CO_2_-sensing cells, and their activity has been implicated in CO_2_ sensing in mammals, frogs, fish, fungi, and plants (Luo et al. 2009; Cummins et al. 2013). Unlike these systems, the BAG neurons of *C. elegans* seem to express low levels of CAs. Out of six predicted α-CAs in *C. elegans*, three lack residues important for catalysis. Only CAH-3 and CAH-4 are active in heterologous CA activity assays (Sherman et al. 2012). Based on its published expression pattern, *cah-4* seems to be absent from BAG (Bretscher et al. 2011), and here we show that *cah-3* is also repressed in these cells. Together, it seems that BAGs do not require high levels of CA activity to fulfill their function. This might reflect the fact that while other systems likely sense HCO_3_^−^ or H^+^, BAGs sense CO_2_ directly (Smith et al. 2013).

### A cell type-specific miRNA carves out the expression of broadly expressed genes

We report that *cah-3* and *akap-1* are normally repressed within the main CO_2_-sensing neurons of *C. elegans*, and their derepression is detrimental to the animal's behavioral response to an important environmental cue. However, because their gene products function in basic cellular biology, *cah-3* and *akap-1* are broadly expressed in all major cell types of the animal. For example, down-regulation of *akap-1* using RNAi causes embryonic lethality and sterility (Maeda et al. 2001). Here we show that a miRNA expressed exclusively in the CO_2_-sensing neurons provides the required specificity to the repression of these genes.

Our findings align with observations that so-called ubiquitous genes are often expressed at different levels in different cell types and can be regulated by external conditions. For instance, β cells in the pancreas rely on the specific repression of two otherwise ubiquitous genes—a lactate/pyruvate transporter (*Mct1*) and lactate dehydrogenase (*Ldha*)—to prevent inappropriate release of insulin when blood glucose is low (Ishihara et al. 1999; Thorrez et al. 2011). While the source of specificity of this repression is unknown, it seems to occur at the transcriptional level. Derepression of *Mct1* has been linked to exercise-induced hypoglycemia in humans (Otonkoski et al. 2007); thus, it is important for coordinating systemic responses of a complex organism with changing internal conditions. Here we show that this type of cell-specific repression is necessary in specialized cells that interact with the environment and that precise repression is provided by a specifically expressed miRNA. It had been shown that a miRNA has the capacity to repress a ubiquitous reporter carrying an artificial 3′ UTR (Mishima et al. 2009). Here we show that this is a naturally occurring strategy for neuronal specialization with impact on a whole organism.

Two properties of broadly expressed genes and miRNAs suggest that this might be a more general strategy. First, transcriptional control of housekeeping genes is generally more compact and constrained than the modular regulation of developmentally regulated genes (Zeitlinger and Stark 2010; Zabidi et al. 2015). This might make transcriptional regulation more difficult. Second, the fast evolution of miRNAs makes it more likely for novel targeting specificity to arise (Berezikov 2011). Overall, post-transcriptional regulation via miRNAs might be a more likely evolutionary solution to cell-specific repression of broadly expressed genes.

Many miRNAs are expressed with high spatiotemporal specificity, perhaps most noticeably in animals like *C. elegans*, where cell types are represented by as little as single cells. For example, the miRNA *lsy-6* is made and acts in a single neuron (Johnston and Hobert 2003; Cochella and Hobert 2012). In addition to *mir-791*, we found at least another 26 miRNAs expressed in single or few cell types (K Mahofsky, T Drexel, and L Cochella, unpubl.). We predict that some of these will also regulate broadly expressed genes in specific contexts. Moreover, a highly specialized site of action, rather than redundancy or fine-tuning modulation, may explain to a large extent why we failed to ascribe functions to many miRNAs.

CO_2_ evokes diverse responses in different animals, ranging from being an attractant when related to food finding to being a repellent when it signals predators or stress (e.g., Cayirlioglu et al. 2008). Therefore, the molecular and neural mechanisms of CO_2_ sensing and CO_2_-evoked behaviors are dynamic over evolutionary time even within different isolates of *C. elegans* (Hallem and Sternberg 2008). We showed that a miRNA can modify this behavior and, given the relatively fast rate of miRNA evolution, propose that *mir-791* and others, such as *mir-279* in *Drosophila* (Cayirlioglu et al. 2008), have played a role in the adaptation to different CO_2_-sensing requirements. Interestingly, while most miRNAs are well conserved among different *Caenorhabditis* species, *mir-791* is relatively divergent. It will be interesting to explore the potential connection between *mir-791*-mediated regulation and the ability of different species to respond to CO_2_.

## Materials and methods

### Strains

All worm strains were grown under standard conditions (Brenner 1974). A full list of strains used here is in Supplemental Table S2.

### Generation of mutant alleles

Protocol, primers, and sgRNA sequences are in the Supplemental Material and Supplemental Table S3.

### Behavioral assays

Behavioral assays were performed using a similar device previously used for O_2_ sensory responses (Zimmer et al. 2009) with modifications (see the Supplemental Material).

### Calcium imaging

Transgenic animals expressing GCaMP6f in the BAG neurons (see the Supplemental Material) were imaged as described previously (Zimmer et al. 2009). We reported and quantified the fluorescence relative to a baseline: *ΔF/F* = *(F − F0)/F0*. *F0* is the mean fluorescence of the lower 15th percentile of all data points in the first 50 sec of recording.

### Fosmid recombineering

Fosmid-based reporters were generated as described previously (Tursun et al. 2009). For details, see the Supplemental Material.

### Microscopy

Quantification of the *cah-3* and *akap-1* GFP-based reporters in the BAG neurons was performed on *Z* stacks through the nervous system with a spinning disc microscope. For details, see the Supplemental Material.

## Supplementary Material

Supplemental Material
