# Supplementary material for: Neuron type-specific miRNA represses two broadly expressed genes to modulate an avoidance behavior in C. elegans
Source: Genes Dev. 2016 Sep 15;30(18):2042–7. doi: 10.1101/gad.287904.116 (PMC5066611; doi:10.1101/gad.287904.116)
Supplement: Supplemental Material [file supp_30_18_2042__index.html]

Neuron type-specific miRNA represses two broadly expressed genes to modulate an avoidance behavior in C. elegans — Supplemental Material 

# Neuron type-specific miRNA represses two broadly expressed genes to modulate an avoidance behavior in *C. elegans*

## Supplemental Material

- Supplemental\_Table\_3.xlsx
- Supplemental\_Methods.pdf
