# Supplementary material for: Neuron type-specific miRNA represses two broadly expressed genes to modulate an avoidance behavior in C. elegans
Source: Genes Dev. 2016 Sep 15;30(18):2042–7. doi: 10.1101/gad.287904.116 (PMC5066611; doi:10.1101/gad.287904.116)

SUPPLEMENTAL METHODS

CRISPR/Cas9 mutant allele generation using antibiotic selection

Based on our experience, CRISPR/Cas9-induced homology-directed repair events were found reliably in stable transgenic lines after injection of the Cas9/sgRNA/repair template mix. This does not mean that non-transgenic animals did not have desired events, but focusing on the stable lines restricted the number of animals we had to screen. To enrich for stable transgenic lines without the need to single out large numbers of transgenic F1s after injection we used antibiotic selection.

20-30 hermaprodites were injected with the respective injection mix containing 50 ng/µl *eft-3*^{prom}*:Cas9sv40:nls:tbb-2* (Friedland et al. 2013), 30-50 ng/µl pIK111 or pRB1017 (*U6*^{prom}*:sgSRNA*) (Katic and Grosshans 2013; Arribere et al. 2014), 50 ng/µl repair templates for homology directed repair, 3 ng/µl *myo-2*^{prom}*:mCherry* and 100 ng/µl of a plasmid expressing a neomycin resistance cassette (pBCN44 from Semple et al. 2012). After a short recovery of ~1 hour at room temperature on OP50 food plates, injected adults were transferred to neomycin containing plates (1 adult/plate). Neomycin plates were prepared as described at http://www.wormbuilder.org/reagents/antibiotic-selection/, however our stock solution concentration was 12.5 mg/ml and we distributed only 300 µl per 5 cm NGM plate. Worms were grown at 25 °C and only transgenic progeny will survive which results in relatively few animals on the plates.

As soon as the F2 transgenic generation is visible all F1 mothers within each plate were pooled and tested with the PCR screening method of choice. The PCR product was Sanger sequenced, which gave us a good estimate of how efficiently cuts close to the sgRNA/s had occurred. Cutting events led to the appearance of double peaks at the side of sgRNA binding and can be further

decomposed by the following online tool : https://tide.nki.nl/ (Brinkman et al. 2014).

From plates where we detected editing events, F2 progeny was either singled out to regular OP50 plates (in case only a few F2 were left on the plate) or pooled (2-3 worms, in case of many survivors) and allowed to self. As soon as the F3 progeny is visible, F2 worms can be screened for the required homology directed repair event, which typically consisted on PCR followed by restriction digestion. All mutant alleles were confirmed by Sanger sequencing and the sequences of the obtained 3'UTR alleles are provided below.

Behavioral Assays

Behavioral assays were performed using a similar device previously used for O_2 sensory responses (Zimmer et al. 2009) with some modifications. One-day old, well-fed adults were transferred from growth plates with OP50 bacteria to 10 cm NGM plate without food. They were then subsequently transferred to a 14 cm NGM assay plate containing an arena (56 x 56 mm) delimited by Whatman paper soaked in 20 mM $CuCl_2$, which prevents the animals from leaving the assay area. 50 to 100 animals were assayed per experiment. Animals were starved for 1 hour prior to recording onset. For each round of experiments, a separate WT and *mir-791(0)* dataset was recorded as controls in parallel with the additional tested genotypes.

Gas flow was applied through a custom made Plexiglas device (60 x 60 x 0.7 mm), which was placed onto the assay arena and connected via a static mixing element to mass flow controllers (Vögtlin Instruments) operated by customized LabView software to control gas concentrations (v/v). Gas flow was maintained at 100 ml/min. For the first 240 sec, animals were exposed to 21% oxygen. At constant 21% oxygen, CO_2 was increased to 5% at a rate of 0.5% / 5 sec. CO_2 concentration was then kept constant for 500 seconds and eventually decreased

back to 0% at the same rate. All gas mixtures were balanced with N_2. Movies were made at 3 frames per second using a 4 Mpixel camera (Jai) streaming to Streampix software. Movies were analyzed with a modified MatLab-based tracking software (Ramot et al. 2008), available at http://med.stanford.edu/wormsense/tracker. For speed calculation only periods of continuous forward movement were used and data were binned by taking the mean of 3 consecutive frames. Reversals and turns were detected by measuring characteristic changes in angular velocity and data were binned by taking the sum of events in consecutive 15 frames intervals.

For traces in Fig. 1B (top) and in Supplemental Figs. S2 and S4, we calculated the population mean, which is the mean across all tracked animals in each bin. For cumulative sum profiles (240-360 s) in all other figure panels we averaged the population means from individual replicates, across all replicates (each replicate is a population assay with 50-100 worms, each dataset consists of ~9-15 replicates for each genotype). Each datapoint shown in the boxplots and used for quantifications, is the total cumulative sum (240-360 s) of the population mean response in each individual replicate. For all behavioral experiments a non-parametric test was applied (Mann-Whitney), using Prism 7.

Calcium Imaging

A transgene expressing GCaMP6f under the BAG-specific promoter from the *flp-17* gene was injected as complex array. We tested 4 of the obtained transgenic lines for behavior in either wt or *mir-791(0)* background. We found that one of the 4 lines recapitulated wild type and mutant behavior, while the other three lines showed dampened behavioral responses in wild type animals, possibly due to the Ca^{2+} buffering effect of GCaMP overexpression (Supplemental Fig. S5A).

Ca^{2+} imaging of BAG neurons was performed using a previously described microfluidic device (Zimmer et al. 2009) used here for delivering CO_2 stimuli.

Animals were imaged in S-basal buffer containing 1 mM of the muscle paralyzing compound tetramisole, to reduce movement. CO_2 concentrations were controlled with a static gas mixer connected to mass flow controllers (Vögtling Instruments) operated by LabView software. Total gas flow at the inlet to the device was set to 50 ml/min. Fluorescence of GCaMP6f expressed in BAG was recorded with a Zeiss Plan-Apochromat 40x/1.4 oil immersion lens on an inverted epifluorescence microscope equipped with a CoolLED pE-2 excitation system (470nm) and Piston GFP bandpass filter set. Imaging data were acquired with a Photometrics Evolve 512 EMCCD camera with 100 ms exposure time, streaming images at 10 Hz acquisition rate to VisiView software. Fluorescence intensity time series were extracted from the image sequences using a custom written script in Metamorph software. A region of interest (ROI) and a neighboring background region in each frame were defined based on thresholding. The cell body position was tracked using the Metamorph track objects function, the total integrated fluorescence intensity of the ROI was determined, and background fluorescence was subtracted in each frame. We report and quantify the fluorescence relative to a baseline: $\Delta F/F = (F-F0)/F0$. F0 is the mean fluorescence of the lower 15 percentile of all datapoints in first 50 s recording time. Mann-Whitney tests were used for statistical analysis.

We would like to highlight that the response we observe for BAG has somewhat different kinetics than others reported in previous work (Bretscher et al. 2011, Hallem et al. 2011, Carrillo et al. 2013). This is most likely due to differences in setup that result in different speed of gas concentration change, gas flow and actual concentration changes, in addition to differences in the calcium indicator used. Previous work used YC3.60 or GCaMP3.0 while we use GCaMP6f which should display faster kinetics. Carrillo et al. actually observe different decay kinetics when they compare YC3.60 vs. GCaMP3.0.

Fosmid Recombineering

Fosmid-based reporters were generated as previously described (Tursun et al. 2009). Primer sequences used to build all fosmids and resulting construct sequences described in this work can be provided upon request. Briefly, the *mir-791* fosmid was generated by replacing the precursor miRNA hairpin by *gfp*. The *gcy-9* fosmid reporter was made by replacing the sequence between exons 1 and 4 by *gfp*, this generates a cytoplasmic GFP but still maintains the large intron and all other potential regulatory sequences up and downstream. Both *cah-3* and *akap-1* fosmid reporters were generated by insertion of a *T2A:gfp:H2B* cassette upstream of the stop codon (a recombineering cassette containing the selection marker *galK* in an intron of *T2A:gfp:H2B* is available). This produces two independent polypeptides (Ahier and Jarriault 2013), an untagged AKAP-1 or CAH-3 and a nuclear localized GFP, while both are under regulation from the same 3'UTR. To generate the 3'UTR mutations, the mutated 3'UTRs were fused to the *T2A:gfp:H2B* cassette by PCR and the resulting cassette was used for recombineering. All fosmids were injected as complex arrays at 10 ng/µl together with sonicated OP50 genomic DNA at 100 ng/µl and either the BAG marker *flp-17^{prom}:mCherry* (or *flp-17^{prom}:NLS:mCherry*) for easy cell identification or the AIY-specific *ttx-3^{prom}:mCherry* for transgenic animal selection.

Microscopy

Quantification of the fluorescent intensities in the BAG neurons from animals carrying the *cah-3* and *akap-1* GFP-based fosmid reporters was performed by taking Z stacks (0.4 µm) trough the nervous system with a spinning disc confocal microscope (PelkinElmerUltraView VoX). In all these animals the nuclei of the BAG neurons was labeled by *flp-17^{prom}:NLS:mCherry.* All analyses were done using Fiji (Schindelin et al. 2012). In the case of the *cah-3* reporter the maximum GFP of the BAG neurons was measured at the plane where the highest mCherry signal was detected and compared to the maximum intensity of the posterior

reference neuron REM within the same animal. Only animals in a dorso-ventral orientation were used for quantification, which allowed us to reliably identify the REM reference neurons due to their positioning anterior of the nerve ring and posterior of the BAGs. For the *akap-1* reporter, brighter expression in the hypodermal cells and in other neurons around the BAGs made it difficult to obtain good dorso-ventral views of these neurons. We therefore selected mosaic animals that still expressed the BAG specific marker (*flp-17^{prom}:NLS:mCherry*) that was co-injected with the *akap-1* reporters, but had visibly fewer GFP-labeled cells around them, and scored animals even when rotated as long as at least one BAG neuron was unobstructed. This meant that most times we scored a single BAG neuron per animal and the RME reference neurons were not visible in every animal. Therefore, we used as a reference one of the hyp5 nuclei in the anterior part of the animal. Hyp5 is the most anterior hypodermal cell and is very reliably identified.

Scoring cell type specific markers

Neurons were scored for presence or absence of the respective neuron specific markers and images were taken under a widefield microscope, Axio Imager.Z2 with sCMOS camera running under Metamorph.

SUPPLEMENTAL REFERENCES

Ahier A, Jarriault S. 2013. Simultaneous Expression of Multiple Proteins Under a Single Promoter in Caenorhabditis elegans via a Versatile 2A-Based Toolkit. *Genetics*.

Arribere JA, Bell RT, Fu BXH, Artiles KL, Hartman PS, Fire AZ. 2014. Efficient marker-free recovery of custom genetic modifications with CRISPR/Cas9 in Caenorhabditis elegans. *Genetics* **198**: 837–846.

Bretscher AJ, Kodama-Namba E, Busch KE, Murphy RJ, Soltesz Z, Laurent P, de Bono M. 2011. Temperature, oxygen, and salt-sensing neurons in C. elegans are carbon dioxide sensors that control avoidance behavior. *Neuron* **69**: 1099–1113.

Brinkman EK, Chen T, Amendola M, van Steensel B. 2014. Easy quantitative assessment of genome editing by sequence trace decomposition. *Nucleic Acids Research* **42**: e168.

Carrillo MA, Guillermin ML, Rengarajan S, Okubo RP, Hallem EA. 2013. O2-sensing neurons control CO2 response in C. elegans. *J Neurosci* **33**: 9675–9683.

Frank F, Sonenberg N, Nagar B. 2010. Structural basis for 5'-nucleotide base-specific recognition of guide RNA by human AGO2. *Nature* **465**: 818–822.

Friedland AE, Tzur YB, Esvelt KM, Colaiácovo MP, Church GM, Calarco JA. 2013. Heritable genome editing in C. elegans via a CRISPR-Cas9 system. *Nat Meth* **10**: 741–743.

Hallem EA, Spencer WC, McWhirter RD, Zeller G, Henz SR, Rätsch G, Miller DM, Horvitz HR, Sternberg PW, Ringstad N. 2011. Receptor-type guanylate cyclase is required for carbon dioxide sensation by Caenorhabditis elegans. *Proceedings of the National Academy of Sciences* **108**: 254–259.

Katic I, Grosshans H. 2013. Targeted Heritable Mutation and Gene Conversion by Cas9-CRISPR in Caenorhabditis elegans. *Genetics* **195**: 1173–1176.

Ramot D, Johnson BE, Berry TL, Carnell L, Goodman MB. 2008. The Parallel Worm Tracker: a platform for measuring average speed and drug-induced paralysis in nematodes. *PLoS ONE* **3**: e2208.

Schindelin J, Arganda-Carreras I, Frise E, Kaynig V, Longair M, Pietzsch T, Preibisch S, Rueden C, Saalfeld S, Schmid B, et al. 2012. Fiji: an open-source platform for biological-image analysis. *Nat Meth* **9**: 676–682.

Seitz H, Tushir JS, Zamore PD. 2011. A 5'-uridine amplifies miRNA/miRNA* asymmetry in Drosophila by promoting RNA-induced silencing complex formation. *Silence* **2**: 4.

Semple JI, Biondini L, Lehner B. 2012. Generating transgenic nematodes by bombardment and antibiotic selection. *Nat Meth* **9**: 118–119.

Tursun B, Cochella L, Carrera I, Hobert O. 2009. A toolkit and robust pipeline for the generation of fosmid-based reporter genes in C. elegans. *PLoS ONE* **4**: e4625.

Zimmer M, Gray JM, Pokala N, Chang AJ, Karow DS, Marletta MA, Hudson ML, Morton DB, Chronis N, Bargmann CI. 2009. Neurons detect increases and decreases in oxygen levels using distinct guanylate cyclases. *Neuron* **61**: 865–879.

Supplemental Table S1. Top 10 *mir-791* targets predicted by TargetScanWorm 6.2.

Gene Name	Conserved Sites	Poorly Conserved Sites
akap-1	**2**	**1**
cah-3	**2**	
hbl-1	**2**	**1**
vab-10	2	
F19C6.3	1	1
unc-2	**2**	
unc-9	**1**	**1**
sdn-1	1	
dhs-13	1	
Y46G5A.29	1	

In bold are the 5 candidates selected for validation (Fig. 5 and Supplemental Fig. S7) based on the presence of multiple binding sites as well as a potential connection with CO_2 sensing or neuronal signaling.

For *akap-1*, the two more conserved sites are conserved in *C. briggsae, C. remanei, C. japonica, C. tropicalis* and *C. sp11* (based on the seed-matching sequence), but they seem to be absent in *C. brenneri, C. angaria* and *P. pacificus.* The less conserved site is only present in *C. elegans.*

For *cah-3,* the two sites are conserved in *C. briggsae, C. remanei, C. brenneri, C. japonica, C. tropicalis* and *C. sp11* (based on the seed-matching sequence), but they seem to be absent in *C. angaria* and *P. pacificus.*

Supplemental Table S2. Strains used in this study

Use	Strain Name	Genotype
Behavior	MLC237	*mir-791 (luc39)*
	MLC239	*mir-790 (luc40)*
	MLC319	*mir-791 (luc39); mir-790 (luc40)*
	MLC610	*cah-3 3'UTR mutant (luc28)*
	MLC397	*cah-3 CDS deletion (luc20)*
	MLC657	*akap-1 3'UTR mutant (luc37)*
	MLC524	*unc-2 3'UTR delta 300 bp (luc27)*
	MLC613	*unc-9 3'UTR mutant (luc30)*
	MLC618	*hbl-1 delta 670 bp (luc32)*
	MLC644	*akap-1 (luc37); unc-2 (luc27) unc-9 (luc30) cah-3 (luc28) hbl-1 (luc35)*
mir-791 expression	MLC56, 64-65	*lucEx43, 51-52 (mir-791:gfp fos, ttx-3^{prom}:mCherry)*
mir-791 rescue	MLC672	*lucEx153 (1.5 kb up:mir-791:1.5 kb down); mir-791(luc39)*
	MLC472-473	*lucEx323-324 (flp-17^{prom}:mir-791:mCherry, unc-122^{prom}:dsRed)*
	MLC476-477	*lucEx327-328 (gcy-8^{prom}:mir-791:mCherry, unc-122^{prom}:dsRed)*
	MLC481, MLC483	*lucEx332-lucEx333 (che-1^{prom}:mir-791:mCherry, unc-122^{prom}:dsRed)*
GCaMP imaging	MLC683	*lucEx445 (flp-17^{prom}:: GCaMP6Fopt, unc-122^{prom}:dsRed)*
	MLC682	*lucEx445; mir-791(luc39)*
	MLC686	*lucEx445; gcy-9 (n4470)*
Cell identity	MLC356	*lucEx247 (gcy-9::gfp^{fos}, ttx-3^{prom}::mCherry)*
	MLC355	*lucEx247; mir-791(luc39)*
	OH2410	*otIs151, ntIs1*
	MLC673	*otIs151; ntIs1; mir-791(luc39)*
	MLC646	*lucEx430 (tax-4^{prom}:gfp:H2B, flp-17^{prom}:NLS:mCherry, gcy-8^{prom}:NLS:mCherry, unc-122^{prom}:dsRed)*
	MLC674	*lucEx430; mir-791(luc39)*
	RJP401	*rpIs7 (gcy-33^{prom}:gcy-33:SL2:gfp, rol-6)*
	MLC675	*rpIs7; mir-791(luc39)*
	ZIM701	*mzmEx438 (gcy-31 genomic:SL2:gfp, unc-22^{prom}:gfp)*
	MLC676	*mzmEx438; mir-791(luc39)*
Target expression	MLC787-789	*lucEx500-502 (akap-1:T2A:gfp:H2B^{fos}, dpy-30^{prom}:mCherry:H2B:tbb-2)*
	MLC791-793	*lucEx504-506 (cah-3:T2A:gfp:H2B^{fos}, dpy-30^{prom}:mCherry: H2B:tbb-2)*
Target reporter assay	MLC649-650	*lucEx433-434 (cah-3:T2A:gfp:H2B^{fos} wt 3'UTR, flp-17^{prom}:NLS:mCherry, gcy-8^{prom}:NLS:mCherry)*
	MLC668-669	*lucEx433-434; mir-791 (luc39)*
	MLC653-656	*lucEx437-440 (cah-3:T2A:gfp:H2B^{fos} mutant 3'UTR, flp-17^{prom}:NLS:mCherry, gcy-8^{prom}:NLS:mCherry)*
	MLC765-766	*lucEx492-493 (akap-1:T2A:gfp:H2B^{fos} wt 3'UTR, flp-17^{prom}:NLS:mCherry, gcy-8^{prom}:NLS:mCherry)*
	MLC767-768	*lucEx492-493; mir-791 (luc39)*
	MLC769, 771	*lucEx494, 496 (akap-1:T2A:gfp:H2B^{fos} mutant 3'UTR, flp-17^{prom}:NLS:mCherry, gcy-8^{prom}:NLS:mCherry)*

Supplemental Sequences: 3'UTR sequences of all 3'UTR mutant alleles generated in this study

cah-3 (luc28):
aattttcccagaaaaaagtttttttttcatttttcaaatttaataccgatacaaaaaGcttcatggtcccaagcaactttt
gtcaaaaatccccgttttcctctgatttcaacacttcaagccagcccgatgtctatttttcttatgtagtaattcgcaatt
cattgcaatattctagttactctgacacgaaacatgggttcccactccacctacaattccaactttgttaacaacatt
gaataaatttttagaatttttttgttcatcaatttttcaataaaaagtgtggggtgtgacgtcacgaagaggaagtagtt
gtgttgtgcaaaatagggtcgtatgagctcaaggggtgacggaacgaaatggaattttggaaagatgtgttatcg
g

akap-1 (luc37):
agtcgactatatttgtccatgcttACCGGTattggttgagtgtgtgttgcgatcgatgttaagagggttgTgTgtta
acaagtatttggaatttttttttttagaaacgattcctgtaccaattctctttaacgtcgatctcatcactcattctaccacc
ccaaccatctctacgaagtatccatttttccatttctcatcatcacaatttgcttttctaccgtttccgactgtttctttttaac
ctgtaacacaaaaagcagtgcgtttcatttttatttgtctccttgctaacctatgtttctgaacctaatcattcattgttgta
tgcatttcacgtagttttacatcccatcttaatctaaatattgcttgggttcaggctgtatactcgcgataatgctctcttct
ttttttttaatttcccaaagtctgaggtggagtacggtagtttgtctgttttcgaagccaatattcccatcctctttcctatcc
ttctttcctttttttggtaatgttattttattttccctctattccttccgaatggTACCatcaaatatcaaaccatcacaatat
agttggctgctttttttcatgcctttaatcgttatagatatctttctagggtagttcacaaaatgtgccgtcgctttgcgcgtt
tattcttgttttcttctgtctctccgtgctcgtttttcatcctcgtctcatttttttatatctcaaaatacattcacagaatgattcc
ttgtatacatgcacagaatggc

hbl-1 (luc32):
ccacgacccgacctagtgcatttttactgtgatcattgcaaaattccatttgatgtaagtttagaatagaaagatttga
ataattgaaaagcaaactatttctaaattgtttcagacccagcaagtattggacagtcatatgagattccacacac
cagggaacccgttcatgtgcagtgactgtcagtatcaggctttcaatgagctaagctttgctctccacatgtaccaa
gccagacaccaataatgaggacgtcggaaacacttcccatagccccttaccctcgtctagtgacccattctgaa
atcggcagatagactttt**agttgtctagttgtccctctagttgtcatttgt**ccctctagttgtcatttgttaaatagccg
aagtgacccaacaacccgcttttgtcccctctactaataaccgttttattattattatcactcaatatttatctttttatgta
cttctttcactgctcccatgtcgtgatttctgatttcacattttccagactatctcgcactttcattctacctcaatacatccc
agctttttttgcatacatctcgatcgataagggggtgcctaataaggatcgtgttattatctagaaccatgtatactggt
ctcattactgtataccttccctggaatcctggtattcgtcacagtcactctaatgactcggcaatagtaactattcagg
tcgatgctcaggatgtcgctcgtgggc

hbl-1 (luc35):
tgaggacgtTcgGtaCCaacacttcccatagccccttaccctcgtctagtgacccattctgaaatcggcagata
gactttcatgtagctggtttaagttttctcttcattttctttaacttatcaatgttcgggcgttaccacttttctaatcatggcc
agtttcttgcacgtacacctcaaccgtccatttccatgaaatctcatacttgttactgttttcttttacctctgatgtcacatt
ttctctctgtctcactttctacctccaaacatattttactacttgtattgaaCGaaataccatatttatGTgcattgttcat
ttacagttttgtactctcagagcgtgttattatctagaagcaattgtatactgttctcagtacatgtagtacctccccccag
aatacttgtttctgttactatgtacccctcttattaacttcgggatatgaaactttttatgtttcattttctattgatttcatttgttt
gtcattttcaagctcctctttccacataagctttaactgcatgtctttcatttttatttatttctattCGttgtttaactatgcac

acatttgtttcatgtttctccagagataactttcccaaattcaagtttgcgccaactcgtgctgctcttttattgtacggtttt
ataacgtttccgtcttgaaatcagagattgtagccgtttttgaaaaggataCTAGTgaatcgtCaccctctagtt
gtcatttgttaaatagccgaagtgacccaacaacccgcttttgtcccctctactaataaccgttttattattattatcact
caatatttatctttttatgtacttctttcactgctcccatgtcgtgatttctgatttcacattttccagactatctcgcactttca
ttctacctcaatacatcccagctttttgccatacat

unc-2 (luc27):
caaccgtttaacaaagcctctccttccaacacattcagctttaccacacattgactccattctttccagccattcctgt
aataccacaattcttgccttgccaccttgccgcctttcatccccctttctgtctatctcagcgatctgtctcgaatatttct
cattagttgcgttcacttctagctcac**ca**cttaaaaaccgttgaatttcagatgggtcttataaaatgtcttcattataa
acaaaattctaccttatgatggattcccatcaaatccagtcgttttcatgtgattttagcctttttctattcctcttttccaat
atgcaataaaaaattcttaaaatctt

unc-9 (luc30):
tgacacaccccaacttcgtagctttttcaatttcttctaacaccagttctcaaattttgatcatgaaatctcactttcgaa
aatcatagaattgtcgtcaatgggtgtagtcgtctttagctgatattctcaattttctgttgctagaaaatatcgaatcat
caactgcatttaaacatccagatcaatggaaactattcacttatcttttCctcatcataattctcttgaaaaccaaaa
ctaaacaattttggagggttttagaagaCTAGTacttttaaacttttgtattgaatcaatttttaaatttctcaattgaa
tacttcaaaaagttgctcaaaactttatcgatccattgtcattttcacctcgagctttttgtcaatttcacatttaattcaatt
gttttctaattttttttgtaaattctatttaccaatttccaaattttctattatcaatttatcaaaactttaatgacgcggttgtc
tttatgaatgttcataactttcaaattattatacctaaatttcctctaaaattaactcccaaaagtcttttgtaattttttctc
caattttattccctttccccccatcctcccacttctccttccttctgtggtttctttcaaatggcatcatgtcttttcaaattac
ctggattcacgtttgtaatatagataatttttgtccttccccccaaactcacttgatacaaaaattaactttctcgccctg
taattcatatttcttccagtttgtcttcatcatccaGGTACCtgttcagttttcaccaaagttttgaaaacggaaccc
acaaactttgccatattttattgtggatttggattatttaatattatttgaaaatgaataaaataacaatttttcattctactt
ctcagtaattttatagtagaggaaaatagcatacttgctttgcaaataaaaacaattaaatagaaaagaaaaatg
gcaaaatatattttttgaaaaactatataaaaaccgtcacaaaatttacagacattttg

SUPPLEMENTAL FIGURE LEGENDS

Supplemental Figure S1. The *mir-791* homolog, *mir-790* does not play a role in CO_2 sensing

A. Representative images of animals carrying the *mir-791* fosmid reporter shown in Figure 1A. An embryo and an adult are shown. The dendrites of the sensory neurons going to the tip of the nose of the animals are marked with a yellow arrowhead. Scale bars = 10 µm. **B.** Alignment of the *mir-791* and *mir-790* sequences in different *Caenorhabditis* species. While both share the seed sequences (boxed in gray), they differ substantially in their 3' ends. **C.** Expression of a *mir-790* fosmid-based reporter. This miRNA is also expressed in just a few neuron pairs including BAG and AFD as can be seen from co-labeling of these cells with known markers. Scale bars = 10 µm. **D-F.** Cumulative sum traces and statistical analysis (done as for Fig. 1B) of the CO_2 responses of wild-type (N2) and *mir-791(0)* animals (**D**, same datasets as in Fig. 1); wild-type (n=11) and *mir-790(0)* (n=12) animals **(E)**; or wild-type (n=11) and *mir-790(0); mir-791(0)* (n=12) double mutant animals **(F)**. Each replicate is a measurement on 50-100 worms. *mir-790* mutant animals do not display any measurable defect in this assay. Animals lacking both *mir-790* and *mir-791* also do not show a stronger defect than animals lacking *mir-791* alone. These experiments show that *mir-791* does not act redundantly with the closely related *mir-790* on the CO_2-sensing pathway. Since *mir-790* is expressed at even higher levels than *mir-791* in RNA sequencing experiments (not shown), this is most likely due to the divergence of both miRNAs at the 3' end, which could result in differential target binding; or alternatively, due to *mir-790* starting with a 5' C rather than U, suggesting that perhaps *mir-790* is not efficiently loaded into miRISC and is thus not a strong repressor (Frank et al. 2010; Seitz et al. 2011).

Supplemental Figure S2. *mir-791* does not affect the speed response to changes in CO_2

Locomotion speed (calculated as in Zimmer et al. 2009) of wild-type, *mir-791(0)* and *mir-791* rescued animals in response to changes in CO_2. Traces show the population averages and shading indicates the standard error of the mean. Experiments used for this analysis are the same as those described in Figure 1B. Statistical analysis was performed on the average speed of each experiment during the 50 sec of stimulation with CO_2 (stim) and the preceeding 50 sec (pre). Mann-Whitney tests were all non-significant (ns).

Supplemental Figure S3. *mir-791* exerts most of its effect through its action in the BAG neurons

A. Transgenic rescue of *mir-791(0)* in the BAG neurons: cumulative sum of the turning events in response to CO_2 (obtained as in Fig. 1B) of wild-type, *mir-791(0)* and *mir-791(0)* animals expressing *mir-791* exclusively in the BAG neurons using the *flp-17* promoter (two independent lines). Box and whiskers plots showing statistics on the endpoints as in Fig 2C. Wild-type: n= 11; *mir-791(0):* n= 10; *mir-791(0), Ex* BAG^{prom}*:mir-791*: n= 6 and 9. **B.** Same as in A but *mir-791* expression is driven with the *gcy-8*^{prom} in the AFD neurons. Wild-type/N2: n=19, *mir-791(0)*: n=21, transgenic line 1: n=4, line 2: n=6, line 3: n=6. **C.** Same as in A but *mir-791* expression is driven with the *che-1*^{prom} in the ASE neurons. Wild-type/N2: n=19, *mir-791(0)*: n=21, transgenic line 1: n=12, line 2: n=4. Each replicate is a measurement on 50-100 worms.

Supplemental Figure S4. *mir-791* is not required for the behavioral response to oxygen concentration changes

A. Turning and reversal rates of an average animal for: wild-type/N2: n=8 or *mir-791(0)*: n=6 animals during oxygen down- and up-shifts (21%-10%-21%). **B.** Statistical analysis of turns and reversals during the oxygen downshift (240-420 sec) was calculated as for Fig. 1B. Each replicate is a measurement on 50-100 worms. **C.** Average speed in the absence of stimulus is indistinguishable between wild-type and *mir-791(0)* animals.

Supplemental Figure S5. *mir-791* is required for a normal physiological response to CO_2 in the BAG neurons

A. Behavioral analysis of animals carrying 4 independent transgenes for GCaMP6F expression in BAG in wild-type (black) or *mir-791(0)* backgrounds. As a calcium-binding protein, GCaMP can affect the intracellular calcium concentration and therefore neuronal activity, making the dose at which GCaMP is expressed an important parameter in these experiments. We generated multiple transgenic lines expressing GCaMP6F specifically in the BAG neurons. Only Line 1 expresses GCaMP at a level compatible with the differential behavioral response between wild-type and *mir-791(0)*. While this line is suitable for imaging experiments, the aberrant response in the other three lines suggests that this behavioral response is quite sensitive to the GCaMP dose. **B.** Heat maps showing the change in GCaMP6f fluorescence over time in the 51 animals (N2) and 46 animals (*mir-791(0)*) upon CO_2 stimulation (average traces are shown in Fig. 4B). **C.** Mean and SEM (left) and heatmap (right) of change in GCaMP6f fluorescence of 39 *gcy-9 (n4470)* mutant animals. Note that *gcy-9* mutants exhibit a low magnitude off response, suggesting a possible GCY-9-independent CO_2-sensing mechanism in BAG.

Supplemental Figure S6. AKAP-1 and CAH-3 are broadly expressed genes

Schematics of the bicistronic fosmid-based reporters used to monitor expression of AKAP-1 **(A)** and CAH-3 **(B)**. A cassette including the T2A viral peptide followed by a GFP:H2B fusion was inserted by recombineering before the stop codon of each respective gene. Representative images of transgenic animals are shown. These animals express in addition a “ubiquitous” *dpy-30^{prom}:NLS:mCherry:tbb-2 3’UTR* reporter. DPY-30 is a component of the dosage compensation machinery as well as a member of the SET1/COMPASS complex. **A.** Expression of *akap-1* can be seen in practically all nuclei except for a few motor neurons. **B.** Expression of *cah-3* is also broad but it is absent from some

hypodermal cells along the body, head and tail as well as the most anterior head muscles. All scale bars = 10 µm. **C.** Schematics of the mutations introduced in the 3'UTRs of *akap-1* and *cah-3* both in the context of the fosmid reporters (for the experiment in Figure 4) and in the context of the endogenous loci (for the experiment in Figure 5). These changes introduce AgeI and KpnI sites in the *akap-1* 3'UTR, and a HindIII site in the *cah-3* 3'UTR.

Supplemental Figure S7. Only *akap-1* and *cah-3* but not the other top candidates are functionally relevant targets of *mir-791*

A-C. Cumulative sum traces and statistical analysis of the CO_2 responses of wild-type animals and animals carrying mutations in the 3'UTRs of *hbl-1* **(A)**, *unc-9* **(B)** or *unc-2* **(C)**. Schematics for the wild-type and mutated 3'UTRs are shown for each candidate gene. **D.** Cumulative sum traces and statistical analysis of the CO_2 responses of wild-type and *mir-791(0)* animals and animals with all five tested mutant 3'UTR alleles (i.e. all *akap-1, unc-2, unc-9, hbl-1, cah-3* 3'UTRs mutated).

For (A) wild-type: n=10, *hbl-1*: n=8; (B) wild-type: n=8, *unc-9*: n=7; (C) wild-type: n=10, *unc-2*: n=8; (D) wild-type: n=13, *mir-791(0)*: n=15, 5xmut: n=15. Each replicate is a measurement on 50-100 worms.

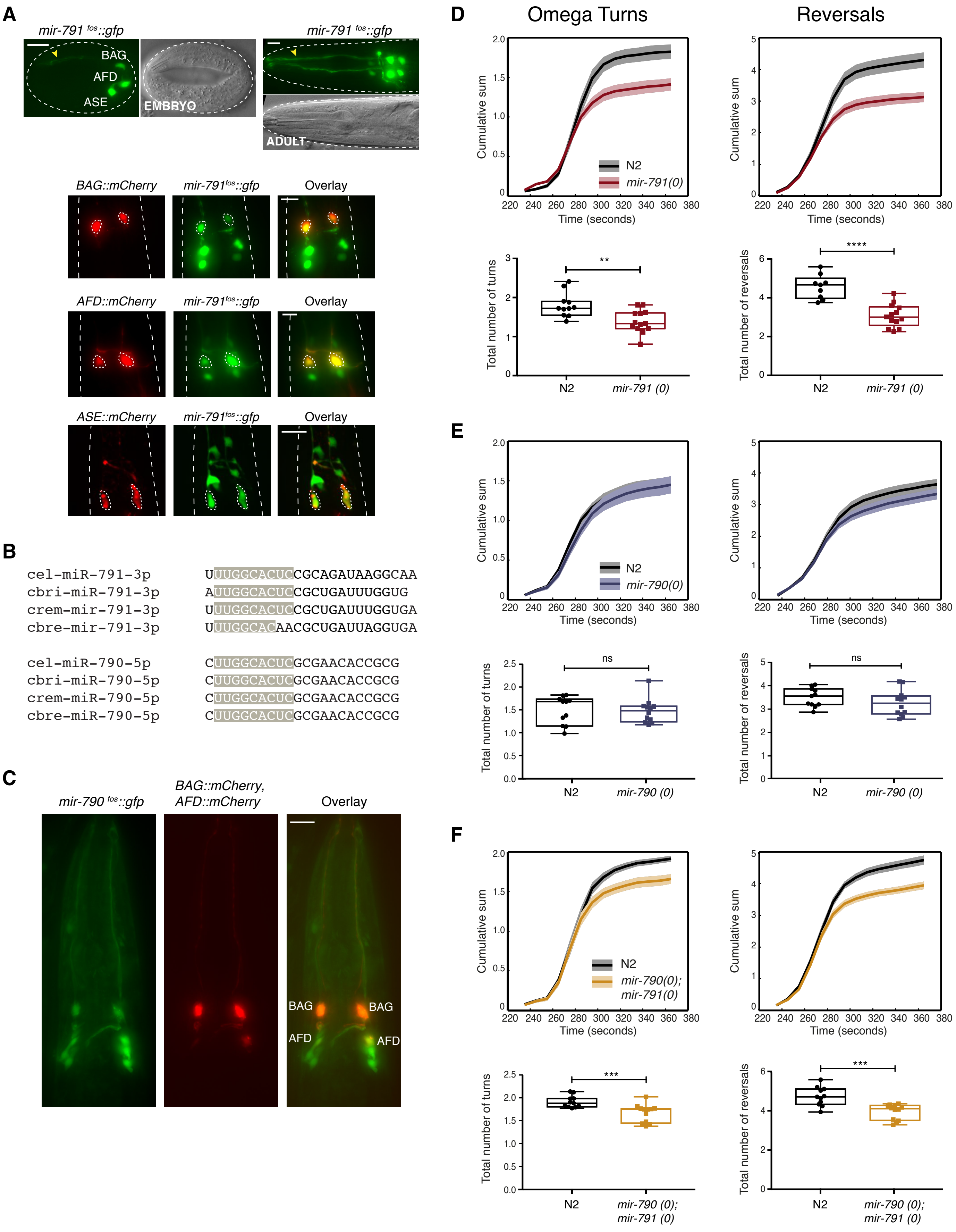
A
mir-791 fos::gfp
BAG
AFD
ASE
EMBRYO
mir-791 fos::gfp
ADULT
BAG::mCherry
mir-791fos::gfp
Overlay
AFD::mCherry
mir-791fos::gfp
Overlay
ASE::mCherry
mir-791fos::gfp
Overlay
B
cel-miR-791-3p UUUGGCACUCCGCAGAUAAGGCAA
cbri-miR-791-3p AUUGGCACUCCGCUGAUUUGGUG
crem-mir-791-3p UUUGGCACUCCGCUGAUUUGGUGA
cbre-mir-791-3p UUUGGCACAACGCUGAUUAGGUGA
cel-miR-790-5p CUUGGCACUCGCGAACACCGCG
cbri-miR-790-5p CUUGGCACUCGCGAACACCGCG
crem-miR-790-5p CUUGGCACUCGCGAACACCGCG
cbre-miR-790-5p CUUGGCACUCGCGAACACCGCG
C
mir-790 fos::gfp
BAG::mCherry,
AFD::mCherry
Overlay
BAG
BAG
AFD
AFD
D
Omega Turns
Reversals
Cumulative sum
Time (seconds)
N2
mir-791(0)
Total number of turns
Total number of reversals
**
****
N2
mir-791 (0)
E
N2
mir-790(0)
ns
ns
mir-790 (0)
F
N2
mir-790(0);
mir-791(0)
***
***
mir-790 (0);
mir-791 (0)

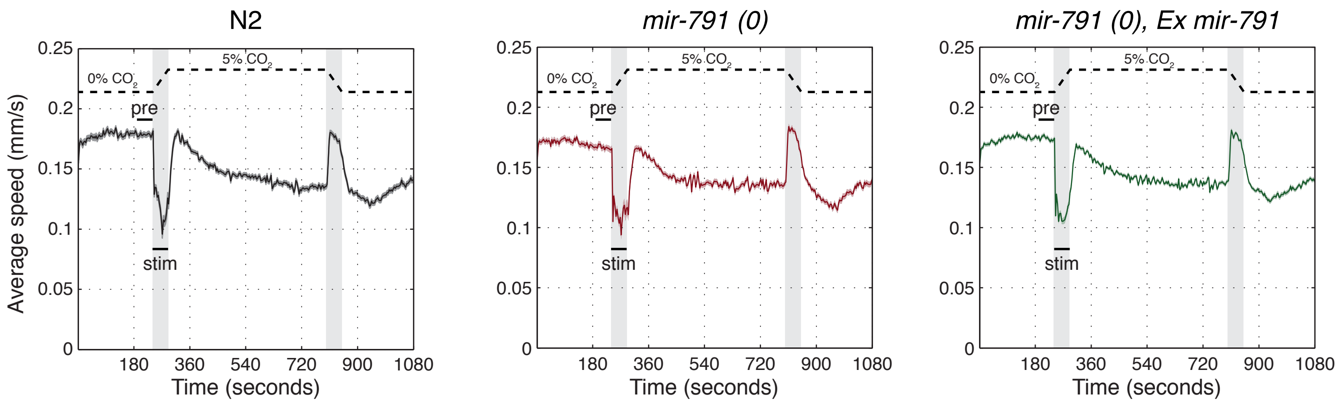
N2
mir-791 (0)
mir-791 (0), Ex mir-791
Average speed (mm/s)
0.25
0.2
0.15
0.1
0.05
0
0% CO_2
5% CO_2
pre
stim
180 360 540 720 900 1080
Time (seconds)

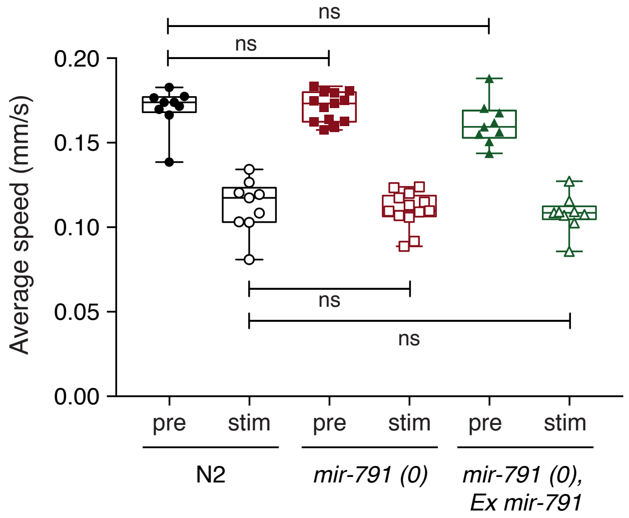
ns
ns
ns
ns
Average speed (mm/s)
0.20
0.15
0.10
0.05
0.00
pre
stim
N2
mir-791 (0)
mir-791 (0), Ex mir-791

Supplemental Figure S3 - Drexel et al.

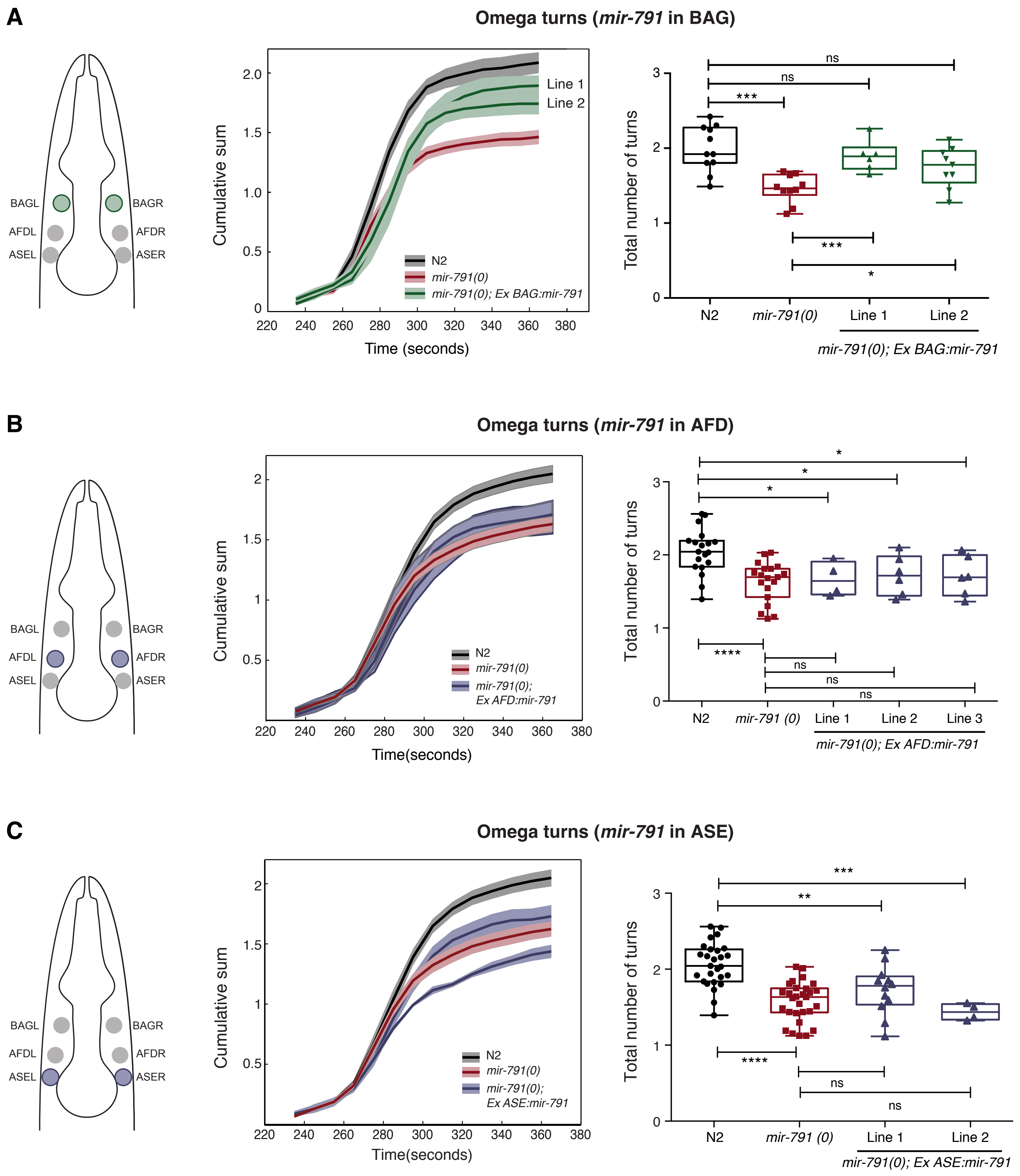

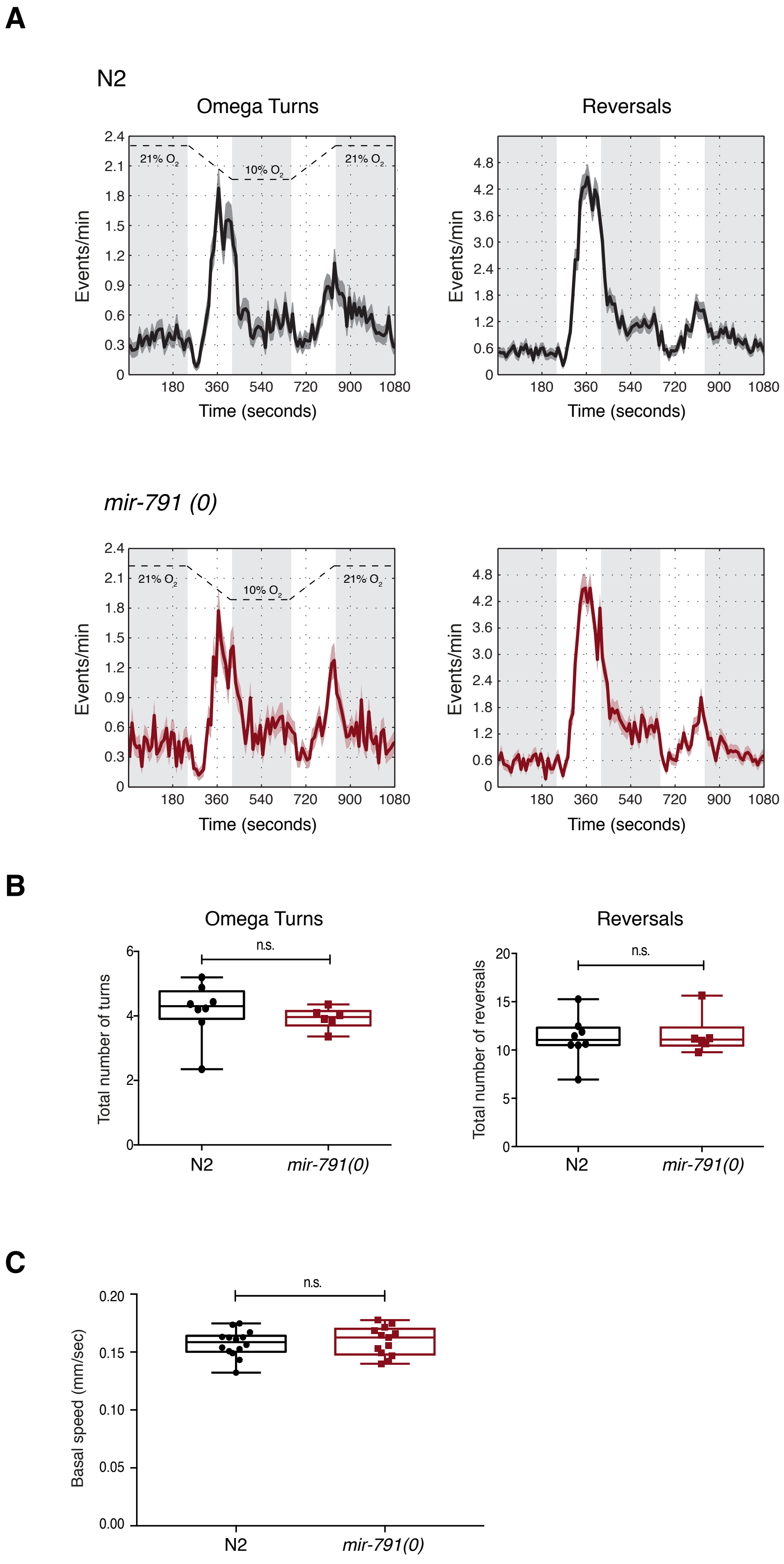
A
N2
Omega Turns
Reversals
21% O_2
10% O_2
Events/min
Time (seconds)
mir-791 (0)
B
Omega Turns
Reversals
n.s.
Total number of turns
Total number of reversals
N2
mir-791(0)
C
Basal speed (mm/sec)

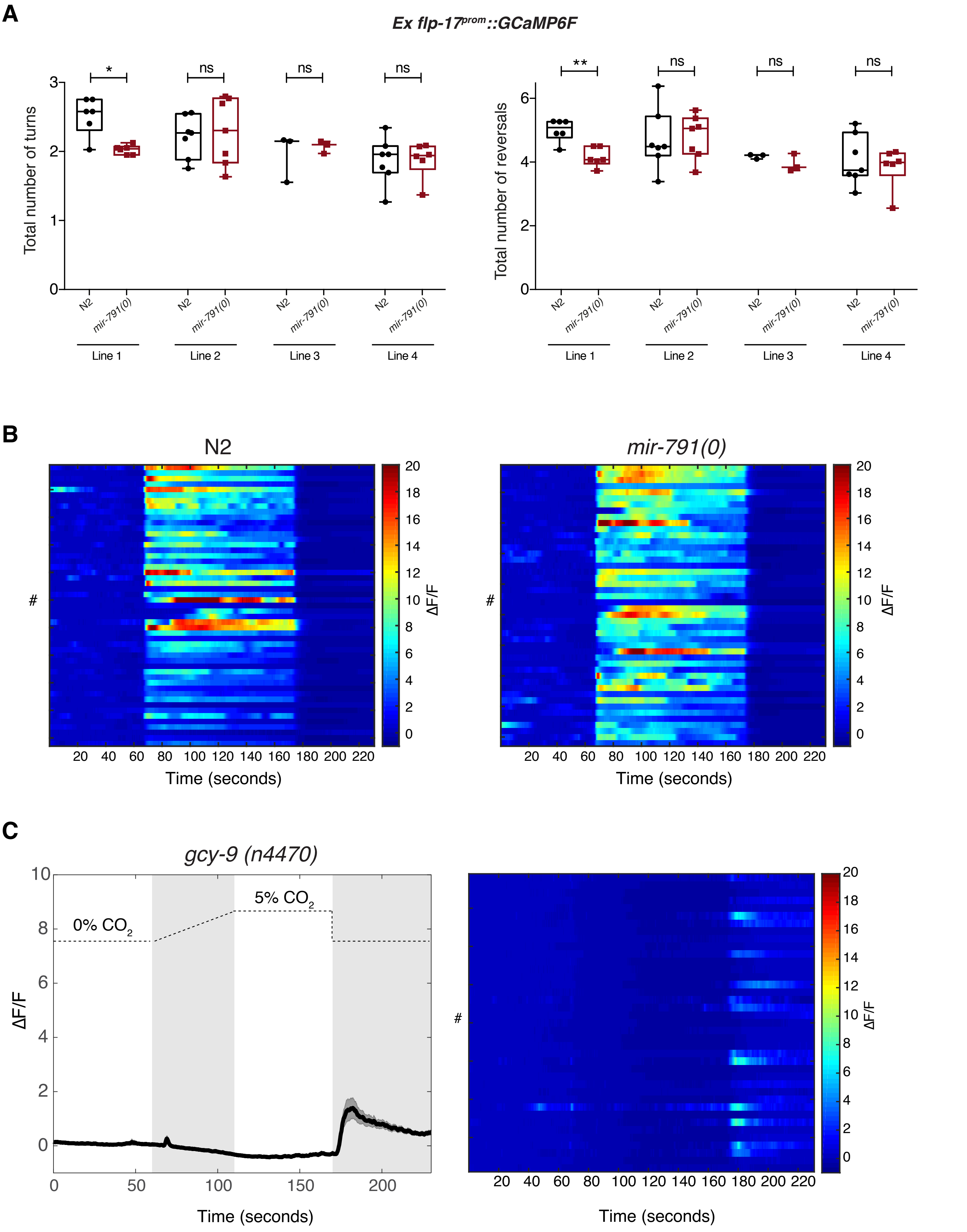
A
Ex flp-17prom::GCaMP6F
Total number of turns
Total number of reversals
*
**
ns
N2
mir-791(0)
Line 1
Line 2
Line 3
Line 4
B
N2
mir-791(0)
#
ΔF/F
Time (seconds)
C
gcy-9 (n4470)
0% CO2
5% CO2
ΔF/F
Time (seconds)

Supplemental Figure S6 - Drexel et al.

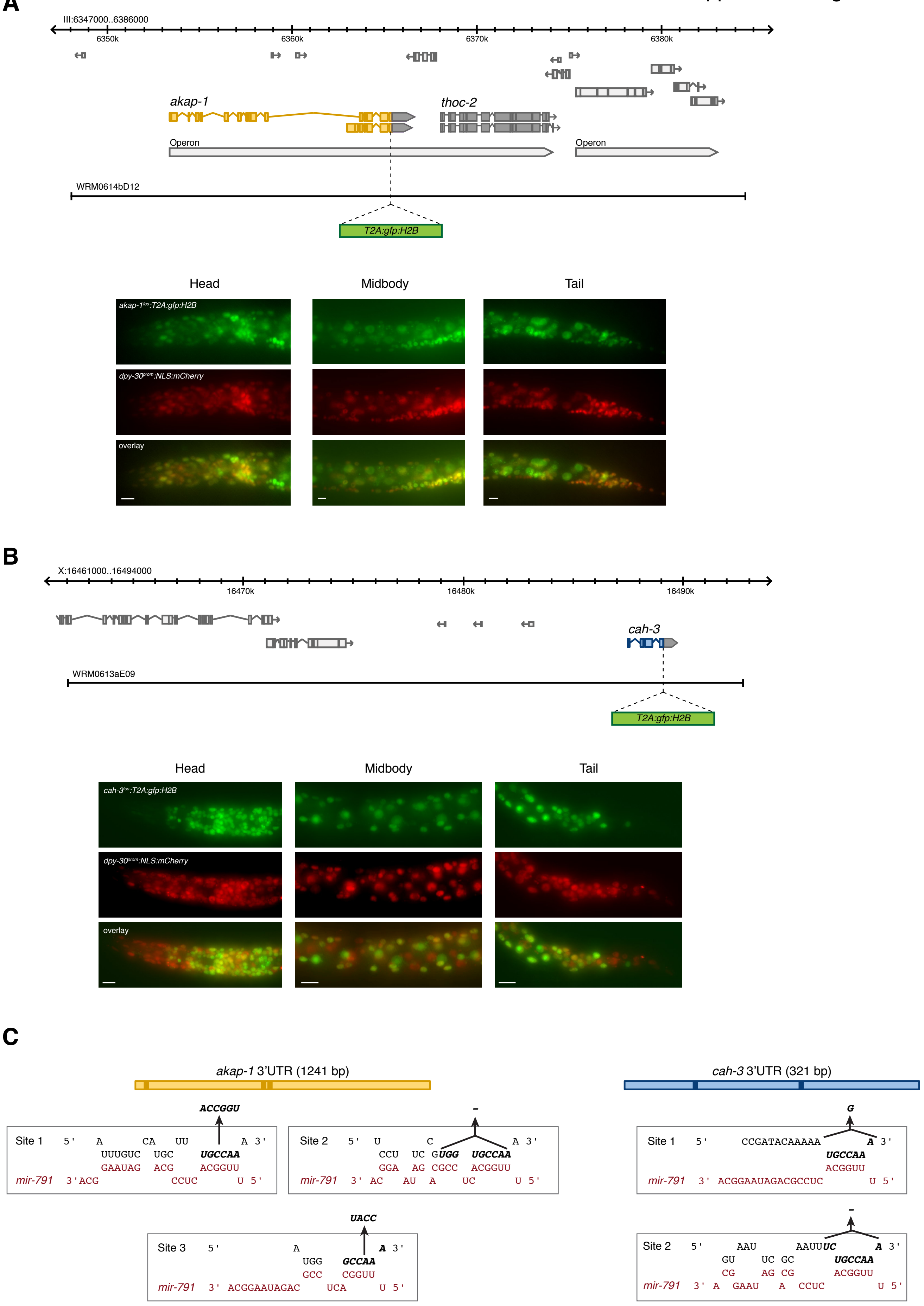

Supplemental Figure S7 - Drexel et al.

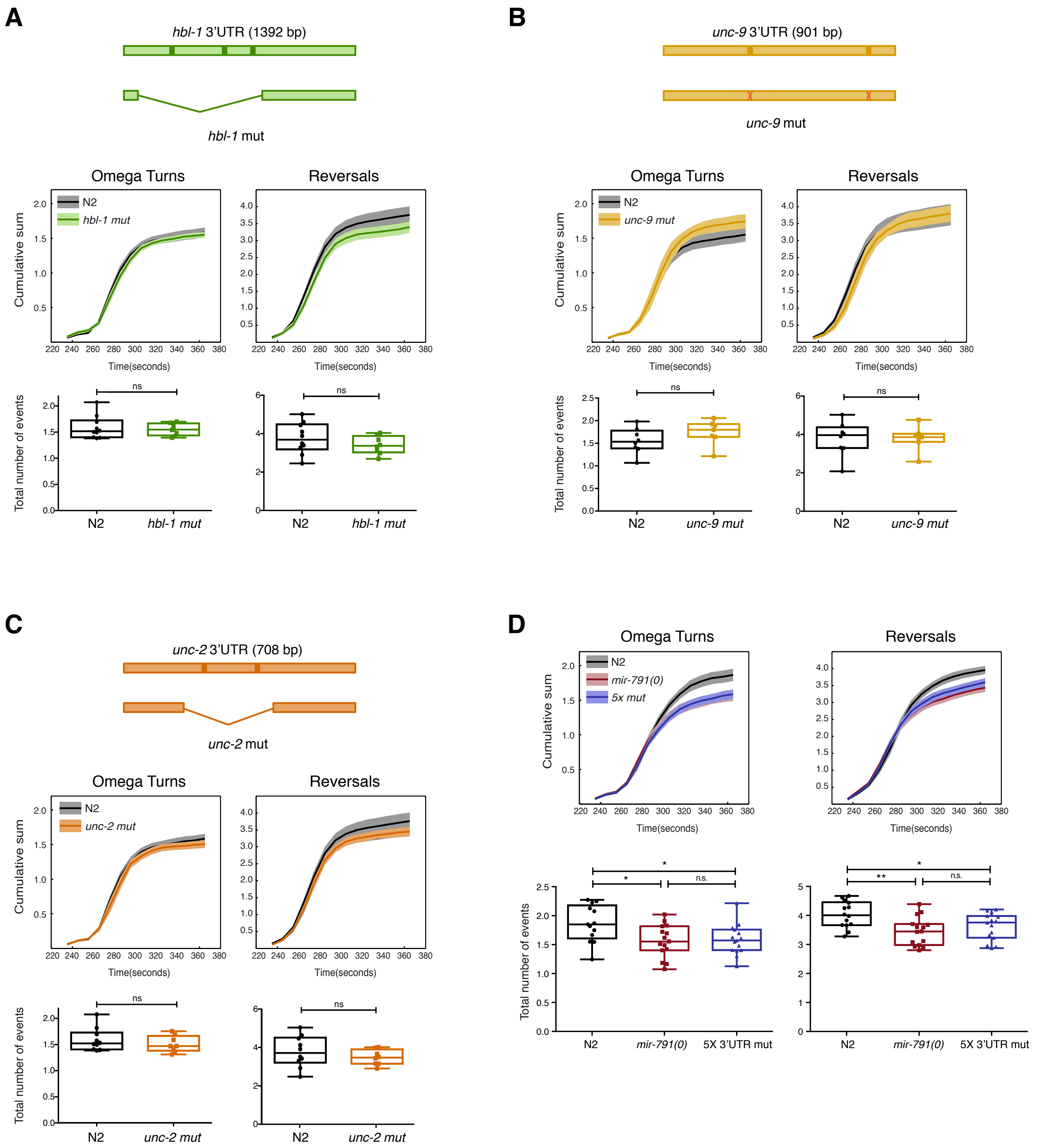

Supplement: Supplemental Material [file supp_gad.287904.116_Supplemental_Methods.pdf]
